# A scalable HPC framework for bioinformatics in resource-limited settings: design principles, implementation, and sustainability from the UVRI experience

**DOI:** 10.1093/bioinformatics/btag149

**Published:** 2026-03-25

**Authors:** Edward Lukyamuzi, Timothy Kimbowa Wamala, Alfred Ssekagiri, Ronald Galiwango, Grace Kebirungi, Atwine Mugume, Mike Nsubuga, Suresh Maslamoney, Sumir Panji, Nicola J Mulder, Daudi Jjingo, Jonathan Kayondo

**Affiliations:** Bioinformatics & Computational Biology (BCB), Uganda Virus Research Institute (UVRI), P.O. Box 49 Entebbe, Uganda; Open Data Science Platform (eLwazi ODSP) for the Data Science for Health Discovery and Innovation in Africa (DS-I Africa); Bioinformatics & Computational Biology (BCB), Uganda Virus Research Institute (UVRI), P.O. Box 49 Entebbe, Uganda; Open Data Science Platform (eLwazi ODSP) for the Data Science for Health Discovery and Innovation in Africa (DS-I Africa); Bioinformatics & Computational Biology (BCB), Uganda Virus Research Institute (UVRI), P.O. Box 49 Entebbe, Uganda; Open Data Science Platform (eLwazi ODSP) for the Data Science for Health Discovery and Innovation in Africa (DS-I Africa); Open Data Science Platform (eLwazi ODSP) for the Data Science for Health Discovery and Innovation in Africa (DS-I Africa); The African Center of Excellence in Bioinformatics and Data-Intensive Sciences, Makerere University, P.O Box 22418, Kampala, Uganda; Open Data Science Platform (eLwazi ODSP) for the Data Science for Health Discovery and Innovation in Africa (DS-I Africa); The African Center of Excellence in Bioinformatics and Data-Intensive Sciences, Makerere University, P.O Box 22418, Kampala, Uganda; Open Data Science Platform (eLwazi ODSP) for the Data Science for Health Discovery and Innovation in Africa (DS-I Africa); The African Center of Excellence in Bioinformatics and Data-Intensive Sciences, Makerere University, P.O Box 22418, Kampala, Uganda; Open Data Science Platform (eLwazi ODSP) for the Data Science for Health Discovery and Innovation in Africa (DS-I Africa); The African Center of Excellence in Bioinformatics and Data-Intensive Sciences, Makerere University, P.O Box 22418, Kampala, Uganda; Open Data Science Platform (eLwazi ODSP) for the Data Science for Health Discovery and Innovation in Africa (DS-I Africa); Computational Biology Division, Department of Integrative Biomedical Sciences, Faculty of Health Sciences, Institute of Infectious Disease and Molecular Medicine, University of Cape Town, Cape Town 7925, South Africa; Open Data Science Platform (eLwazi ODSP) for the Data Science for Health Discovery and Innovation in Africa (DS-I Africa); Computational Biology Division, Department of Integrative Biomedical Sciences, Faculty of Health Sciences, Institute of Infectious Disease and Molecular Medicine, University of Cape Town, Cape Town 7925, South Africa; Open Data Science Platform (eLwazi ODSP) for the Data Science for Health Discovery and Innovation in Africa (DS-I Africa); Computational Biology Division, Department of Integrative Biomedical Sciences, Faculty of Health Sciences, Institute of Infectious Disease and Molecular Medicine, University of Cape Town, Cape Town 7925, South Africa; Open Data Science Platform (eLwazi ODSP) for the Data Science for Health Discovery and Innovation in Africa (DS-I Africa); The African Center of Excellence in Bioinformatics and Data-Intensive Sciences, Makerere University, P.O Box 22418, Kampala, Uganda; Bioinformatics & Computational Biology (BCB), Uganda Virus Research Institute (UVRI), P.O. Box 49 Entebbe, Uganda; Open Data Science Platform (eLwazi ODSP) for the Data Science for Health Discovery and Innovation in Africa (DS-I Africa)

## Abstract

**Motivation:**

Building and sustaining High-Performance Computing (HPC) infrastructure for bioinformatics research in resource-limited settings presents significant technical, financial and operational challenges. Institutions in low-and middle-income regions often face constraints such as limited technical expertise, unstable infrastructure and restricted funding which can hinder the deployment of large-scale computational platforms necessary for modern genomics and bioinformatics analyses.

**Results:**

We present a scalable and modular HPC framework developed at the Uganda Virus Research Institute (UVRI) to support large-scale genomics and other omics data analyses in resource-limited settings. The framework integrates open-source HPC management tools, infrastructure automation, and reproducible configuration management to enable reliable deployment and maintenance. Optimized storage and networking configurations combined with a phased capacity-building strategy support high-throughput genomic workflows while strengthening local technical expertise. From our implementation experience, we derive ten practical design and operational rules that provide a transferable methodology for establishing and sustaining in-house HPC infrastructure. These rules emphasize strategic investment in human capacity, structured planning, leveraging collaborations, adoption of open-source technologies and service management practices to improve operational resilience and long-term sustainability.

**Availability:**

The design principles, automation strategies and implementation guidelines described in this work are applicable to institutions seeking to establish sustainable HPC resources for bioinformatics research in resource-constrained environments.

## 1. Introduction

High-Performance Computing has become an indispensable tool for scientific research that enables researchers to perform complex computations and process large datasets. In the realm of human health, fields such as genomic epidemiology, phylogenetics, and image-based diagnostics leverage big data making the computational capabilities of HPC infrastructure essential to cutting-edge research for discovery and the development of disease treatment or control solutions. However, establishing and maintaining HPCs in resource-limited environments can be daunting. While utilizing commercial HPC services, such as AWS ParallelCluster, Azure HPC and Google Cloud HPC (Jackson et al. 2010, Rodrigues 2023, Dogukan et al. 2025) seems like a viable alternative, unreliable internet access, limited local technical support and difficulties in adapting these services to specific research requirements often render this approach impractical. Consequently, establishing in-house HPC infrastructure not only becomes essential but also an opportunity to create sustainable and context-specific solutions. It’s worth noting that, even though not focused on bioinformatics, some efforts had been made to build capacity in HPC on the continent (Abiona et al. 2011, Amolo 2018; Johnston et al. 2024). However, the development of such infrastructure is often impeded by insufficient technical expertise, infrastructural deficits and inadequate funding.

**Figure 1:**
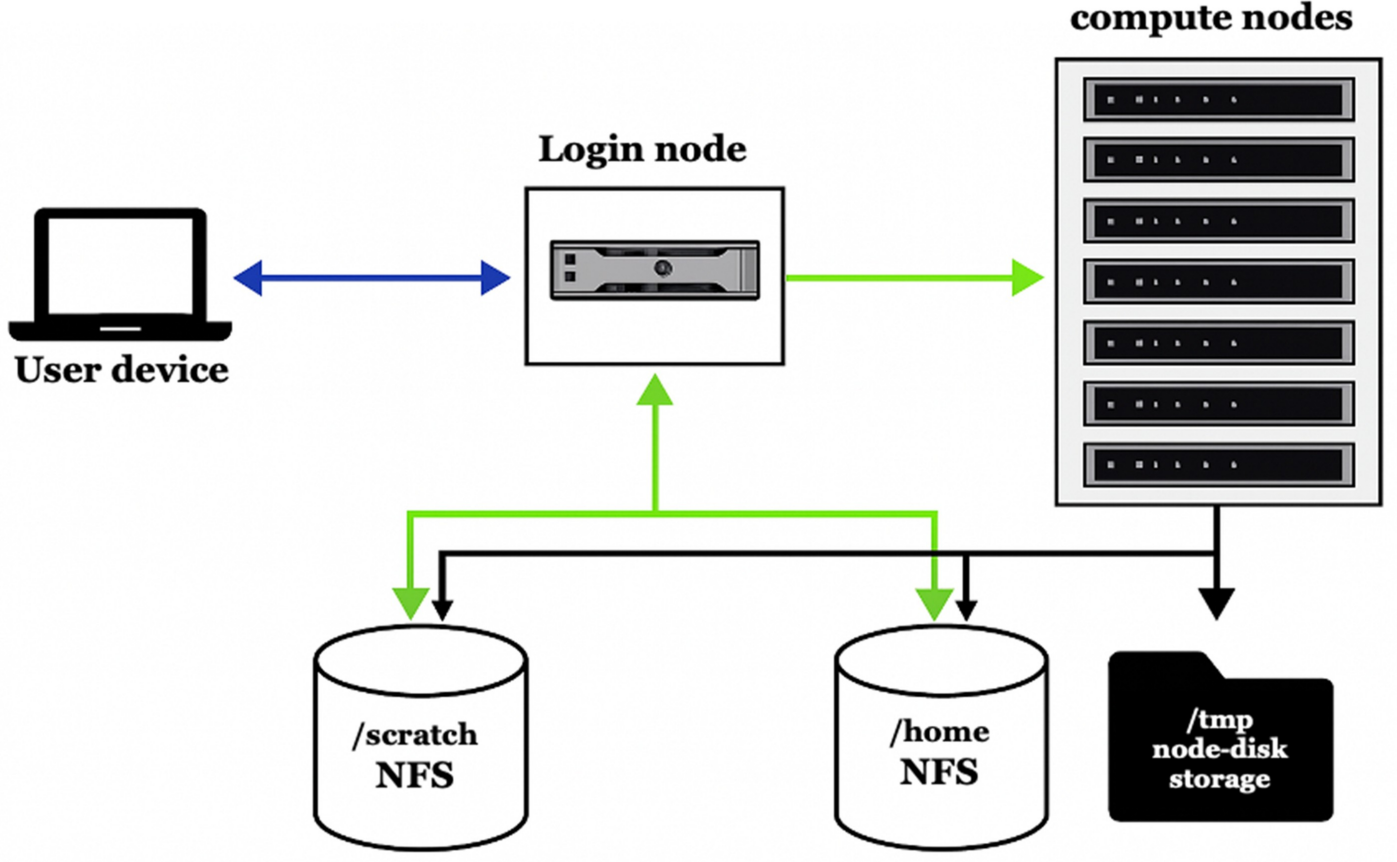
Architecture of the UVRI HPC cluster. Users connect from their devices via SSH to the login node (blue arrow), which provides access to compute resources and shared storage. The compute nodes execute jobs scheduled from the login node and have access to shared /*home* and /*scratch* directories via NFS (green arrows) as well as local /*tmp* storage on each node. This setup enables efficient job submission, shared data access and modular scaling.

The Uganda Virus Research Institute (UVRI) faced many of these challenges when it embarked on a journey to build an HPC cluster. The initiative was driven by the need to develop pathogen and vector genomic surveillance applications, and to facilitate genetic R&D studies for novel vector control tools and pathogen discovery. UVRI also aimed to establish a bioinformatics resource (Fig. 1) capable of supporting the growing genomics needs of the scientific community at the institute and in the region. With start-up hardware and technical capacity upgrade from H3ABioNet (Mulder et al. 2016) together with a donation of servers from the Francis Crick Institute (https://www.crick.ac.uk) through the Makerere University/UVRI Infection and Immunity Research Training Programme—MUII (https://www.muii.org.ug/), we were able to overcome two major start-up hurdles: high capital costs and a local skills gap. These inputs removed the financial, infrastructural and technical‑expertise barriers that usually delay first‑time HPC deployments in low-resource settings.

However, long-term sustainability requires more than donated hardware; it also depends on reproducible automation, risk management and structured service delivery. The framework design and implementation were guided by four complementary principles (modularity, automation, reproducibility, and sustainability) each addressed through dedicated subsystems and management practices. Together, these elements ensure that the cluster can be efficiently deployed, scaled, and maintained within the operational constraints typical of resource-limited settings. The cluster was provisioned using OpenHPC (Baird et al. 2023) for baseline installation and package management with Ansible (https://docs.ansible.com/) playbooks used to automate configuration tasks such as user setup, Slurm (Yoo et al. 2003) scheduler tuning and NFS (Osadzinski 1988) mounts.

## 2. Risk management

Effective service provision requires anticipating operational risks. We implemented a lightweight risk-register model following FitSM (https://www.fitsm.eu/) principles, identifying major risk; hardware aging, power instability, data loss, staff turnover and funding volatility and corresponding mitigations summarized in Table 1.

**Table 1:** Sample risk register summarizing key operational risks in HPC management and the corresponding mitigation strategies for sustainable cluster operation in resource-limited settings.

Risk	Description	Likelihood	Impact	Mitigation strategy
Hardware aging	Donated or legacy equipment may fail more frequently due to age and lack of warranty	Medium	High	Maintain spare nodes and critical parts, schedule proactive hardware refresh cycles every 3–4 years, implement continuous monitoring with alerts
Power reliability	Grid instability and voltage fluctuations can interrupt service or damage components	High	High	Deploy dual mains + solar input with UPS and surge protection, maintain automated shutdown and restart scripts; periodic maintenance
Data loss or corruption	Failure of disks, network, or human error during updates	Medium	High	Use mirrored storage (RAID), off-site backups, and automated snapshot verification; document restoration procedures
Staff turnover/limited expertise	Departure of trained personnel disrupts operations	Medium	Medium	Crosstrain staff, maintain updated system documentation and runbooks, mentor replacements before exit
Funding and sustainability	Irregular project funding jeopardizes maintenance and expansion	Medium	High	Establish cost-recovery model, institutional co-funding, and periodic grant proposals; demonstrate impact metrics to leadership

Power reliability is addressed through dual mains and solar feeds with UPS buffers; data loss risk through mirrored storage and offsite backups and staff turnover risk through continuous cross-training and documentation. FitSM’s structured service management approach also informs our change control, incident logging and policy documentation, helping to institutionalize procedure beyond individuals.

To embed risk awareness into daily operations, we aligned the register with FitSM service-management principles, which emphasize documenting known risks, assigning ownership, and reviewing mitigation actions during change-control cycles. This structured linkage between operational risk tracking and service management ensures that sustainability planning becomes an ongoing, auditable process rather than a one-time assessment.

## 3. Automation and infrastructure as code

To enhance reproducibility and reduce manual configuration errors, automation formed a core design principle. We employed Ansible for configuration management ensuring that installation steps from network setup to Slurm queue definitions, could be executed through declarative scripts. Each node’s configuration is maintained under version control, allowing rollback and peer review of changes. For monitoring and provisioning, we integrated Ganglia (Massie et al. 2004) and Prometheus (Barrett et al. 2023, Sanches and Pereira 2026) dashboards that automatically register new compute nodes once provisioned through the playbooks. Although OpenHPC automates base deployment, the additional Ansible layer enables some sort of software-defined infrastructure approach. Future iterations will extend this to include Terraform templates to describe hardware resources and enable hybrid scaling into cloud environments.

## 4. Ten rules for building and sustaining HPC infrastructure

**Figure 2:**
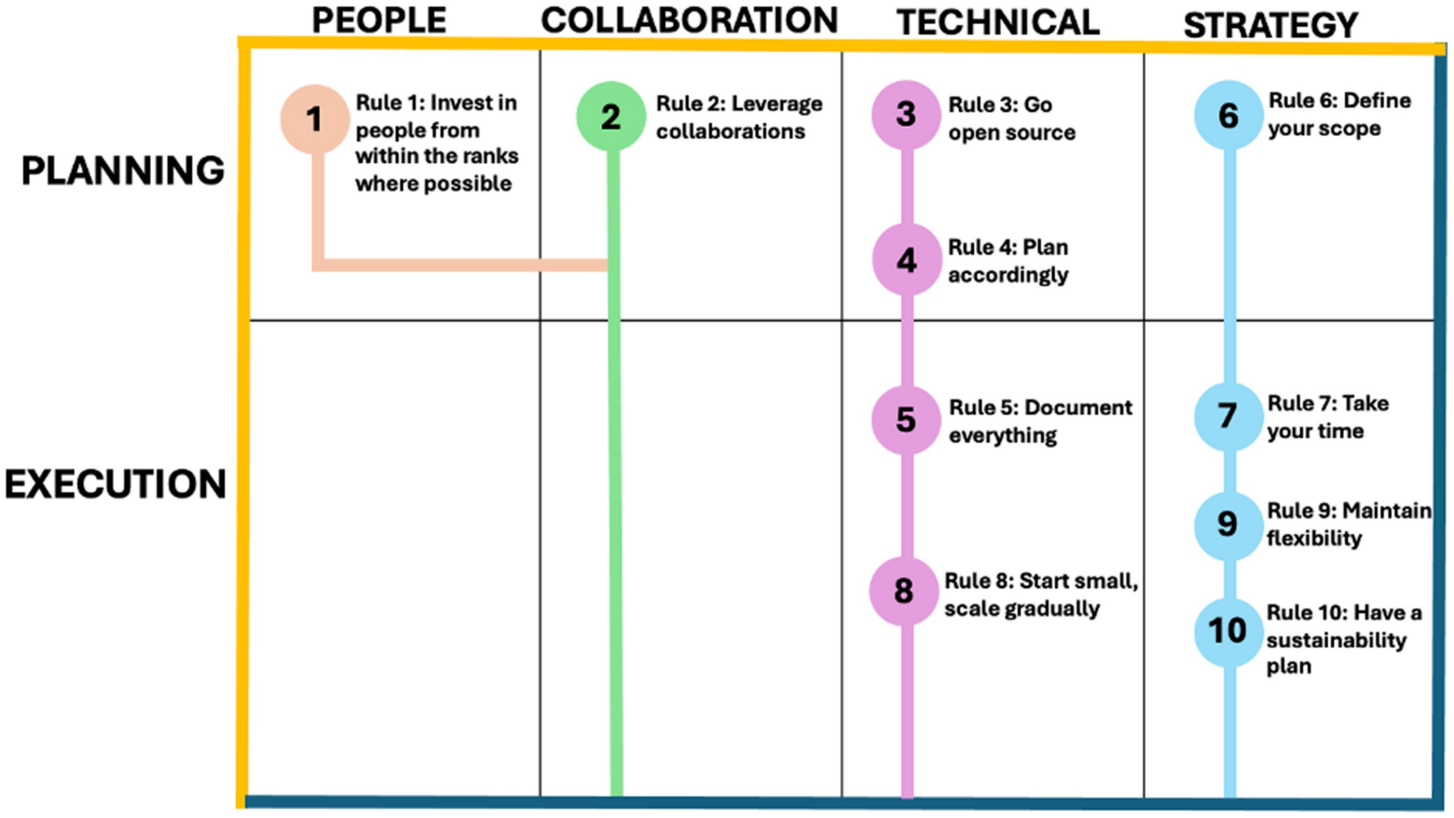
Conceptual map of 10 practical rules for building and sustaining an HPC cluster as a bioinformatics resource in resource-limited settings. Rules are categorized across four thematic domains: people, collaboration, technical and strategy and positioned along two implementation phases: planning and execution. This framework reflects lessons learned from UVRI’s experience, emphasizing local capacity, collaboration, open-source, flexibility and sustainability.

In this article, we share an account of the process, and ten simple rules derived from our experience in building and sustaining an HPC cluster. These principles are organized across two implementation phases: planning and execution and grouped by four domains: people, collaboration, technical and strategy (Fig. 2). These rules are not only applicable to institutions in low-income settings but can also serve as practical guidelines for any organization looking to build and sustain HPC infrastructure. From strategies for investing in human capital and leveraging collaborations to adopting open-source technologies and developing a sustainability plan, we provide an account of the steps we took and the lessons we learned along the way. Our aim is to provide a blueprint for other research institutions facing similar challenges to guide them in building HPCs that are functional, scalable and sustainable.

### 4.1. Rule 1: Invest in people from within the ranks where possible

The backbone of any successful HPC project is the people behind it. Institutions should develop a deliberate capacity-building plan with clearly defined roles and progressive skill levels. A typical structure distinguishes between (i) system administrators who manage hardware, networking, and schedulers, (ii) software or workflow engineers who containerize tools and maintain pipelines, and (iii) scientific users who run analyses and interpret results. Each stream requires tailored learning objectives. Training should combine formal courses (e.g. HPC Carpentry, PRACE training portal, EuroCC materials) with applied mentorship inside the organization. Apprenticeship models work well—junior staff shadow experienced administrators during deployments before managing small subsystems independently. Internal hackathons, documentation sprints, and “train-the-trainer” programmes reinforce peer learning and expand institutional teaching capacity.

Competence tracking is equally important. Periodic evaluations can include measurable indicators such as number of production incidents resolved, workflows containerized, or training sessions delivered. These metrics help justify institutional investment and demonstrate maturity to funders. When external expertise is required, aim for short technical exchanges that transfer skills rather than outsourcing routine administration.

At UVRI, we adopted a home-grown capacity-building strategy, focusing on developing a core team of well-trained individuals from within our existing staff rather than recruiting skilled experts. While this approach takes longer, it ultimately decreases reliance on expensive consultants while building local capacity to sustain efforts even when project funding ceases. This includes providing technical training and encouraging staff to continuously expand their knowledge through workshops, reading, and engaging with the broader community. It’s important to make these activities part of their deliverables. Attending seminars, technical meetups, and hands-on workshops enhances expertise and ensures that the HPC cluster evolves with the latest trends and technologies. Institutions should treat this as an ongoing investment, where nurturing local talent pays off in long-term operational stability and innovation. Additional context, including the full UVRI use-case for Rule 1, is presented in supplementary data (section S1.1).

### 4.2. Rule 2: Leverage collaborations

In resource-constrained settings, collaboration may be the lifeline that transforms an ambitious plan into a sustainable reality. Partnerships can provide access to equipment, expertise and support that would otherwise be beyond reach. Establishing collaborations should be intentional and multi-layered, balancing immediate technical gains with long-term knowledge exchange. Institutions can pursue three complementary forms of collaboration: (i) technical and infrastructural support—Partner with organizations upgrading or decommissioning hardware (e.g. universities or research centers in the Global North). However, donated hardware often carries risk and, in most cases, no warranty. Before accepting equipment, document service life, warranty status, and maintenance obligations. It is critical to record such risks in a register to pre-empt failures and plan mitigations. (ii) Knowledge and community networks—Join regional and continental research computing communities such as RSSE-Africa (https://rsse.africa/), H3AbioNet Infrastructure working group and HPC Ecosystems project. These networks provide mailing lists, configuration templates, and shared policies that accelerate deployment. Shared documentation repositories or “infrastructure-as-code” examples are especially valuable to new sites. (iii) Collaborative service models—Formalize collaborations through Memoranda of Understanding (MOUs) or consortium frameworks that define resource sharing, training exchanges, and data-management expectations. Adopting frameworks like FitSM can help structure service agreements with clear ownership, support boundaries, and escalation procedures. Effective partnerships also require reciprocity. Host institutions should contribute back by maintaining shared scripts, documenting lessons learned, or mentoring new members in the network. Over time, this transforms the collaboration from donor-recipient into a peer community of practice. From our experience, partnerships are vital not just for what they provide in the short term, but for the long-term knowledge exchange and community support that sustains HPC operations over time. See Supplementary Section S1.2 for the complete UVRI collaboration case study related to Rule 2.

### 4.3. Rule 3: Go open source

Open-source software presents a compelling option for organizations in resource-constrained environments as it offers dual advantage: it is cost saving and offers access to vibrant supportive communities. A practical open-source strategy should address four dimensions: selection, integration, support, and contribution. (i) Selection: adopt mature, well-supported projects. Choose components with active user communities and regular releases. Core examples include OpenHPC for system provisioning, Slurm for workload management, and XDMoD (Palmer et al. 2015) for usage monitoring. Evaluate new tools against reproducibility, community health, and security-update cadence rather than novelty. (ii) Integration: combine tools into a coherent, documented stack. Use automation frameworks, for example, Ansible, Terraform (https://www.terraform.io/) to deploy consistent environments. Containers such as Singularity/Apptainer (Kurtzer et al. 2017), Docker (Merkel 2014) encapsulate dependencies while workflow engines [Nextflow (Di Tommaso et al. 2017), Snakemake (Köster and Rahmann 2018, Mölder et al. 2021), WDL (https://openwdl.org)] standardize analysis pipelines. Version-control all configuration files in Git (Chacon and Straub 2014) to ensure traceability and easy rollback. (iii) Support: plan for the lack of commercial on-call help. Open source solutions while powerful also carry the risk of limited formal support. Mitigate this risk through community engagement: join discussion lists, contribute bug reports, and maintain internal troubleshooting logs. Collectives such as RSSE-Africa, HPC Ecosystems, OpenHPC forum, serve as informal help desks. (iv) Contribution: feed improvements back. Even small code patches, translated documentation, or benchmarking data strengthen the ecosystem and raise institutional visibility. Encourage staff to present configuration insights at HPC meetings or submit pull requests to upstream projects.

At UVRI, choosing open-source solutions was driven by financial considerations and the need to stay at the forefront of cutting-edge technology. While the lack of expensive licensing fees was an initial draw, the long-term benefits went far beyond cost. Ultimately, our decision to embrace open-source solutions allowed us to build a flexible, cost-effective cluster that could grow and adapt over time. It also reinforced our belief in collaborative community-driven development which shall help sustain our HPC project into the future. A more detailed UVRI use-case relevant to this rule is available in Supplementary Section S1.3.

### 4.4. Rule 4: Plan accordingly

The success of any infrastructure deployment hinges on thorough planning, with the end-goal in mind. It’s not enough to secure the right hardware; you also need to account for the environmental and operational conditions that will support it. For instance, do you have reliable power and cooling systems? What about expertise to manage the cluster? It’s essential to anticipate these from the outset. HPC deployments succeed when planning is systematic, risk-aware, and aligned with institutional research goals. Planning must consider scientific needs, environmental constraints, and operational readiness—not just hardware acquisition. In resource-limited settings, oversights in power, cooling, staffing, or data policies can jeopardize the entire investment.

Effective planning involves four interlinked components: needs assessment, environmental readiness, architectural design, and risk-informed decision-making.

1. Conduct a structured needs assessment. Before purchasing hardware or provisioning software, institutions should identify: expected workloads (e.g. WGS, RNA-seq, machine learning, genome assembly), required storage capacity and data-retention policies, anticipated computational demand (cores, memory, GPUs), user groups and their skill levels and compliance requirements (data security, ethics, national regulations). Surveys, interviews, or lightweight workload modelling help quantify these needs.
2. Evaluate environmental readiness. HPC clusters depend on stable infrastructure. Institutions should assess: power stability, availability of backup systems, and voltage protection, cooling capacity and room airflow, network performance, internal bandwidth, and internet reliability, physical security and access control. Where gaps exist, mitigation strategies should be incorporated into the implementation roadmap.
3. Use a modular architectural planning approach. Adopt a design process that documents requirements, constraints, and design decisions in a design baseline file. This includes: node architecture (CPU/GPU/memory profiles), storage layers and growth plan, network topology and switch port allocation, scheduling configuration (queues, partitions, fair-share policy), monitoring and backup strategy. Using modular, standards-based components (OpenHPC, Ansible, Slurm) ensures the system can evolve without major redesign.
4. Plan with risks and sustainability in mind. Planning should be grounded in a risk register (see Section 2). For each design choice, institutions should evaluate: hardware aging and replacement cycles, staff availability and training needs, future expansion requirements, procurement constraints and warranties, impact of power or network interruptions. Frameworks like FitSM help map these risks to formal processes (change control, incident management).

At UVRI, we spent significant time understanding our technical infrastructure needs as well as the capacity of our IT team and end users. Planning also involves knowing who will be using the cluster and for what purposes to ensure that the design meets those specific research demands. We conducted an extensive review of the types of research being conducted at the institute, including that coming in from collaborators, and the kinds of projects the HPC would need to support. These included projects related to genomics, bioinformatics, epidemiology and other computationally intensive domains. Our goal was to design a system that would meet not just the needs of current users but could scale to accommodate future research directions as well. A crucial part of our planning was developing a business plan that outlined clear objectives, milestones, a phased-implementation approach and potential areas for expansion. The phased approach (discussed in detail in rule 8) allowed us to implement the cluster in stages, ensuring that each phase was feasible within our available resources while planning for future growth. A detailed UVRI use-case illustrating this rule is provided in Supplementary Section S1.4.

### 4.5. Rule 5: Document everything

Proper documentation is vital for ensuring continuity especially in environments with high staff turnover or limited technical expertise. It extends beyond recording technical setups, it is also about sharing experiences, challenges and solutions. Effective documentation must be systematic, structured, and maintained as part of daily operations rather than produced ad hoc during crises. A practical documentation strategy should cover five pillars: policies, procedures, configuration, troubleshooting, and audits. (i) Policies (the why and who)—Define acceptable use, data-retention rules, storage quotas, backup schedules, access levels, security practices, and escalation procedures. Frameworks such as FitSM or ITIL-lite (Fry 2010) offer templates for service definitions, incident categories, and change processes. (ii) Procedures and runbooks (the how)—These include step-by-step guides for onboarding users, creating accounts, resetting passwords, deploying new nodes, updating software, or recovering from failures. Runbooks reduce load on administrators and make operations repeatable. (iii) Configuration documentation (the what)—Use Infrastructure-as-Code (IaC) (Ansible, Terraform, Slurm configs) stored in a version-controlled repository such as GitHub (https://github.com/) or GitLab (https://gitlab.com/gitlab-org/gitlab-ce). Include design baselines, such as network maps, firewall rules, storage layouts, so that redeployment at a new institution is straightforward. (iv) Troubleshooting, incident logs, and monitoring. Institutions must maintain structured logs for system alerts, failures, performance anomalies, and user issues. Platforms like GitLab Issues, Redmine (https://www.redmine.org) or Jira (www.atlassian.com) can track incidents, categorize root causes, and document solutions. This transforms documentation from static text into an operational feedback system. (v) Documentation sustainability—Assign clear ownership (e.g. a documentation lead), run quarterly reviews, and keep a change log tied to configuration updates. Automate documentation generation where possible using JupyterBook (Jupyter et al. 2025), MkDocs (https://www.mkdocs.org/user-guide/writing-your-docs/), or Sphinx (https://www.sphinx-doc.org/en/master/). This structured approach transforms documentation from a passive record into an active operational control.

At UVRI, we saw this as essential to maintaining the operational stability of our HPC cluster. By maintaining thorough records of our infrastructure, processes, and policies, we created a robust institutional memory. This ensures that even if key personnel leave, the knowledge they have gained remains within the organization, reducing the risk of operational disruptions and costly downtime. When new staff members or researchers join UVRI, they can refer to our comprehensive documentation to quickly familiarize themselves with the system, its policies and best practices. This reduces the learning curve and allows new team members to become productive much faster. Additionally, as new technologies and software are introduced to the cluster, we intend to update the documentation to ensure that the system remains relevant and continues to meet the needs of the user community. All technical configurations were stored in Git, and an internal GitLab tracker logged issues and solutions. A JupyterBook-based documentation hub serves as a single point of truth for both users and administrators. A detailed UVRI-specific case study illustrating the implementation of this rule is provided in Supplementary Section S1.5.

### 4.6. Rule 6: Define your scope

HPC resources are finite, especially in resource-limited settings. Without a clearly defined scope, who the system serves; which workloads are supported and how resources are allocated, clusters become overburdened, misused, or unable to meet strategic objectives. Clear boundaries are a helpful tool to mitigate this. Defining scope ensures fairness, sustainability, and alignment with priorities.

A robust scope definition consists of four key components: a service catalogue, eligibility criteria, prioritization framework, and governance structure.

1. Create a service catalogue (what the HPC provides). A service catalogue defines the boundaries of the HPC service. It should specify supported workload types (e.g. WGS analysis, molecular surveillance, machine learning); supported software stacks and workflow engines; storage tiers (hot, warm, archival), retention periods, and transfer mechanisms; available computational queues (short, medium, long jobs; GPU queue if applicable) and user support levels.
2. Define eligibility and fair usage (who gets access and under what rules). Institutions should establish eligibility rules based on: institutional affiliation, alignment with organizational mission or national research priorities, compliance with data security and ethics policies, and availability of funding or service-level agreements Fair-usage policies should define limits on CPU-hours, storage quotas, and job priorities. Clear expectations reduce conflict and improve planning.
3. Use a transparent prioritization framework (how decisions are made). To avoid subjective or politically motivated allocation, use a criteria-based process such as: scientific impact/public health relevance, data volume and computational demand, feasibility and readiness of the project, availability of funding or co-support, and collaborative value (capacity building, mentoring potential). A small steering committee should periodically review project requests using a standardized scoring rubric.
4. Build modular expansion plans. Scope evolves over time. Institutions should maintain a roadmap for adding storage or compute nodes, introducing specialized hardware (e.g. GPUs), adapting to new scientific directions and revising user categories and quotas. This ensures that growth aligns with actual demand.

We implemented a structured scope definition process through (i) a service catalogue describing supported genomics, molecular surveillance, and data science workloads; (ii) eligibility rules prioritizing high-impact pathogen and vector surveillance projects; and (iii) fair-usage policies defining queue limits and storage quotas. A steering group reviews new project requests, assessing public health relevance, computational requirements, and collaboration value. This framework allowed the cluster to remain focused on strategic research priorities while supporting regional collaborations without overcommitting resources. See supplementary section S1.6 for the extended UVRI case narrative relevant to this rule.

### 4.7. Rule 7: Take your time

Building an HPC particularly in resource-limited settings is a time-consuming process and unrealistic deadlines often lead to suboptimal solutions. At UVRI, we adopted a patient approach, consulting widely and deeply at every stage. This allowed us to gather feedback, assess various options and ultimately select solutions tailored to our specific needs and constraints. Institutions should adopt an incremental deployment model that emphasizes learning, validation, and risk reduction. A practical approach could include four components.

1. Begin with a minimal viable cluster (MVC). Start with a small number of nodes (e.g. 2–5) to validate networking and storage performance, scheduler configuration, authentication, security, and monitoring, automation scripts and IaC consistency and basic user workflows and pipelines. This controlled environment allows timely iteration.
2. Follow an iterative deployment cycle (Plan → Test → Review → Scale). Each expansion cycle should include: Plan—define the upgrade goal (e.g. more cores, faster storage, better cooling), Test—prototype configurations in the MVC or a staging environment, Review—document outcomes, failures, bottlenecks, and lessons learned and Scale—apply changes to production only after validation. This process reduces configuration drift and avoids ad hoc changes.
3. Conduct post-implementation reviews After each deployment or change: assess system stability and user experience, review monitoring metrics (CPU usage, I/O, failures), update risk register and documentation, and adjust training or policies if new challenges emerged. Such reviews institutionalize continuous improvement and increase resilience.
4. Allow time for capacity development. A slow, deliberate timeline gives system administrators time to understand system behavior under real workloads, gradually master advanced configuration, build confidence in troubleshooting and develop automation and documentation. Fast deployments often outpace staff development, leaving institutions dependent on external support.

UVRI adopted a phased rollout strategy, starting with a 5-node cluster used to build baseline skills in job scheduling, monitoring, and storage management. Before adding nodes or upgrading networking, administrators conducted test deployments, documented lessons, and validated changes against real workloads. Each phase included a review of risks, training needs, and configuration updates. By scaling only after gaining operational confidence, we avoided costly misconfigurations and built a stable HPC environment capable of supporting diverse bioinformatics workloads. Additional context is presented in Supplementary Data (Section S1.7).

### 4.8. Rule 8: Start small, scale gradually

Excitement about having a large, high-powered cluster can sometimes lead to overambitious plans. Large HPC deployments are expensive to build, maintain, and operate. Starting small allows institutions to tailor the system to real workloads, prioritize essential functions, and avoid overinvestment in underutilized resources. Gradual scaling ensures that infrastructure growth is driven by genuine demand and operational readiness. A successful scale-up strategy should be data-driven, modular, and aligned with clearly defined service priorities. Three practices help ensure efficient, sustainable growth.

1. Build a modular architecture that supports incremental expansion. Use hardware and software choices that allow components to be added without major redesign. Modularity includes node-based scaling (commodity servers, homogeneous or heterogeneous), expandable storage arrays (RAID, ZFS, Ceph), flexible networking (10/25/40 GbE or InfiniBand switches with free ports), containerized workflows that operate independently of hardware profiles.
2. Use real demand metrics to guide expansion decisions. Instead of guessing future needs, institutions should track usage and bottlenecks using monitoring tools (e.g. XDMoD, Prometheus, Grafana) including CPU-hours consumed, parallel job concurrency, storage utilization trends, I/O patterns (burst vs. sustained loads), queue wait times and failure rates. Scale only when ample periods (e.g. 3–6 months) of monitoring indicate sustained pressure on resources.
3. Expand in defined phases with documented milestones For each expansion cycle: set measurable objectives (increase storage by 30%, reduce queue wait times by 40%); add a small number of nodes or disks, validate, then proceed; update documentation, automation playbooks, and risk register; re-evaluate service catalogue and user priorities as capacity grows. Gradual expansion ensures technical stability and avoids overwhelming limited staff capacity.

**Figure 3:**
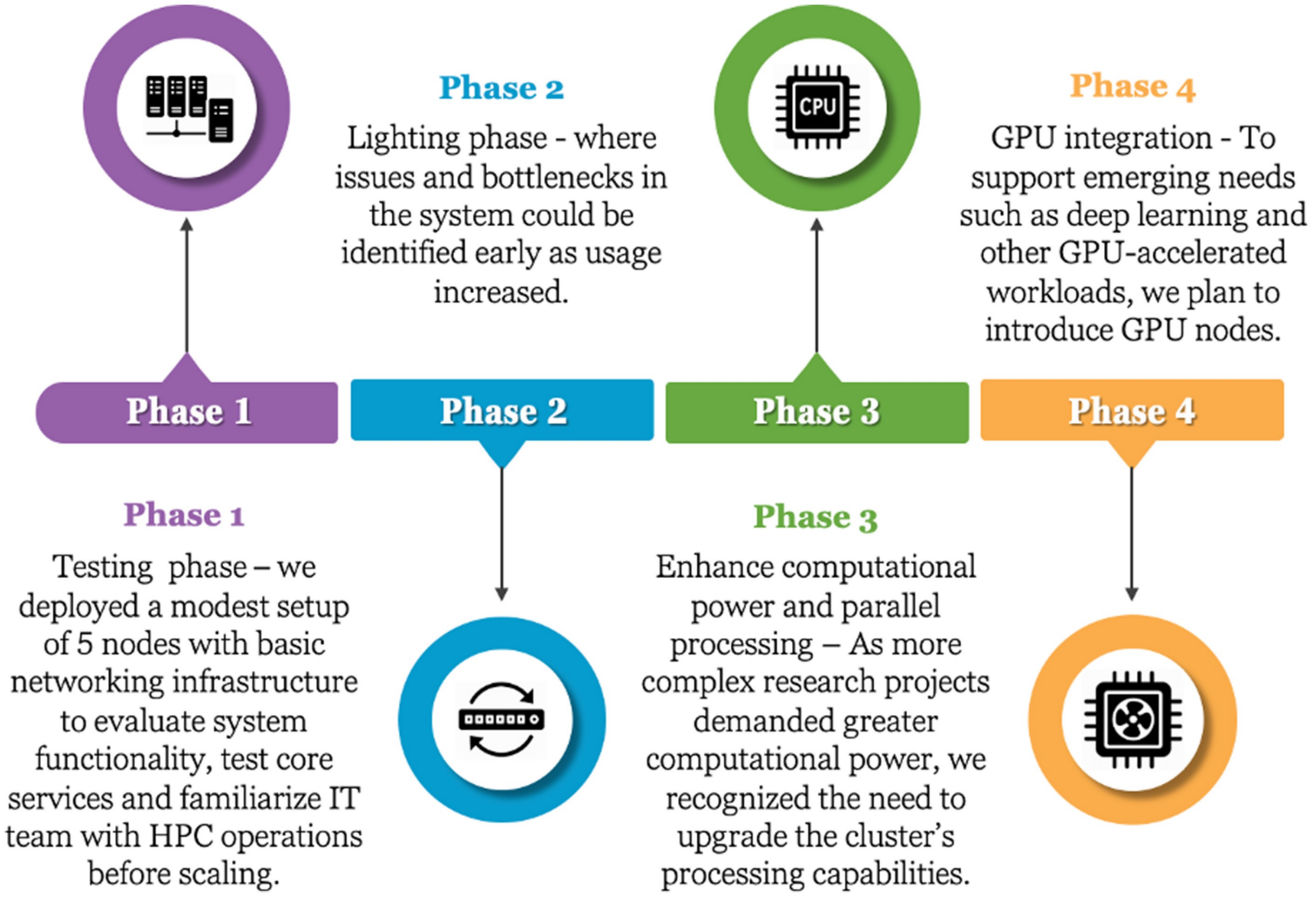
HPC growth strategy at UVRI. This diagram illustrates our four-phase approach to developing HPC infrastructure. The strategy began with a testing phase (Phase 1) using a modest 5-node setup to establish core functionality and build team capacity. Phase 2 involved fine-tuning the infrastructure in response to growing demand, including network upgrades and improved storage. In Phase 3, the focus shifted to enhancing computational power through gradual CPU upgrades for improved parallel processing. Phase 4 anticipates the integration of GPU nodes to support advanced workloads such as deep learning and image analysis.

Starting small allows for careful testing, learning and scaling as needed. At UVRI, we started with a small cluster of 5 nodes and used that initial setup to identify our actual computational needs. This approach not only reduced upfront costs but also provided a lighting phase where we could identify challenges and fine-tune the infrastructure. Starting small allows for phased growth where you can scale the cluster in a modular way based on research demands and funding availability. By adopting this strategy (Fig. 3), we could make targeted upgrades over time to ensure each component of the system aligned with user requirements and available resources. See Supplementary Section S1.8 for our complete case study related to Rule 8.

### 4.9. Rule 9: Maintain flexibility in infrastructure and operations

Building an HPC cluster in a low-resource setting requires a flexible mindset. The needs of your user base, technological advancements and funding sources can shift over time and your infrastructure should be able to adapt to those changes. Flexibility must be engineered into both the infrastructure and the operational processes that support it. We propose four approaches to help institutions remain responsive and future-proof.

1. Adopt portable and modular software environments. Scientific workflows change rapidly. Using tools that separate software from hardware helps maintain stability: Containers (e.g. Singularity/Apptainer, Docker) allow reproducible environments independent of node configuration; Workflow engines (Nextflow, Snakemake, WDL) enable pipeline portability across clusters or cloud resources; Module systems [e.g. Lmod (Geimer et al. 2014)] organize multiple tool versions and avoid conflicts. These tools let the infrastructure evolve without breaking user applications.
2. Keep architecture adaptable to new technologies. Design the cluster so components can be upgraded or replaced without full redesign: leave free network switch ports for future compute or storage nodes, use storage systems that can expand incrementally: ZFS (https://openzfs.github.io/openzfs-docs/), Ceph (Weil et al. 2006), RAID (Patterson et al. 1988) expansions; plan for future GPU or high-memory node integration, choose chassis and power systems that allow mixed hardware generations. This reduces the cost of adopting emerging technologies.
3. Ensure operational flexibility through documented processes. Technical flexibility is ineffective without matching operational agility: use change-management processes (FitSM/ITIL-lite) to evaluate risks and impacts before modifying production systems, maintain staging environments for testing updates, implement rolling updates to avoid full downtime and use incident logs to refine procedures and operational readiness. Operational processes should support experimentation while protecting system stability.
4. Support hybrid and collaborative workflows. Allowing interoperability with cloud or partner HPC systems increases resilience and collaboration potential: use common workflow languages and container formats, maintain compatible authentication systems [e.g. SSH (Ylonen 2006) keys, OpenID Connect (https://openid.net/specs/openid-connect-core-1_0-final.htm)], explore hybrid scheduling (local Slurm + cloud bursting where connectivity allows) ad share documentation and code with partner sites for cross-deployment consistency. This flexibility is especially important when local infrastructure faces outages or funding delays.

At UVRI, we quickly realized the importance of maintaining flexibility in both our technical setup and our operational processes. This meant choosing modular infrastructure components that could be easily upgraded and keeping our operational workflows open to adjustments as new technologies and demands emerged. This adaptability enabled the cluster to support evolving research needs, from SARS-CoV-2 surveillance to large-scale malaria genomics, without major reconfiguration. Flexibility ensures that your HPC cluster can evolve alongside the research it supports to remain valuable and efficient over the long term. A detailed UVRI use-case relevant to this rule is available in Supplementary Section S1.9.

### 4.10. Rule 10: Have a sustainability plan

Sustainability should be baked into the DNA of any HPC project. It’s about more than just keeping the lights on, it’s about understanding your environment, your user base and the scope of your operations. It involves benchmarking against other institutions, especially established ones while tailoring plans to fit your local ecosystem. Without an explicit sustainability plan, even well-designed clusters risk degradation or abandonment once initial project funding ends.

A sustainable HPC strategy must address four pillars: financial planning, operational continuity, institutional embedding, and community engagement.

1. Establish a financial model that supports long-term operations. Sustainability requires predictable funding for: hardware replacement and upgrades (typically every 3–5 years), storage expansion, power, cooling, and network infrastructure, support for essential software, backups, and security instrumentation and staff training and retention. Possible financial models include: institutional co-funding (the most stable), cost-recovery schemes (small fees per project or analysis), hybrid project-based funding, national or regional shared HPC budgets. The plan should include a depreciation schedule and rolling upgrade roadmap.
2. Preserve operational continuity through redundancy and documentation. A sustainable cluster can withstand both technical and personnel changes: maintain redundant hardware (spare disks, power supplies, and standby nodes), use tiered backups and off-site storage for critical datasets, keep regularly updated documentation aligned with FitSM change and incident management, ensure multiple staff can perform key administrative tasks (avoid single points of failure), maintain an actively updated risk register. These measures protect the cluster against unforeseen disruptions.
3. Embed the HPC into institutional structures. Long-term sustainability requires that the HPC become a recognized institutional service, not a side project: assign formal roles (Service Owner, Process Manager, HPC Lead), include HPC metrics (uptime, usage, training delivered) in annual reports, align the HPC roadmap with institutional strategic goals, encourage leadership to champion the HPC as a core research asset. Embedding ensures the cluster remains relevant and visible to decision-makers.
4. Build and nurture a community of practice. Clusters thrive when knowledge flows outward and inward: participate in regional communities, share tools, scripts, and SOPs publicly, host local workshops, hackathons, and training sessions, encourage users to contribute back through documentation or code. These activities strengthen local ownership and reduce dependency on external consultants.

At UVRI, we recognized that sustainability was about more than just keeping the system operational, it required a multi-faceted strategy balancing between technical capacity, financial planning and community engagement. We benchmarked against other successful HPC initiatives with intent to tailor a sustainability model that would work within our specific context, considering the long-term technical and operational upkeep of the HPC infrastructure. We also focused on understanding our user community’s needs, skills and potential, ensuring that our infrastructure was built to support them in a meaningful, lasting way. Annual budgets cover power, cooling, and hardware refresh cycles, while partnerships provide training opportunities and peer support. Documentation, risk registers, and automation scripts ensure operational continuity. A key part of our sustainability plan was bringing on board other UVRI groups, which was made possible by having a conceptualized development plan in place and demonstrating our initial capacity on the ground. This created buy-in and showed the broader organization that the infrastructure could support not just genomics and bioinformatics research, but also other computationally intensive domains. The HPC is now embedded as a core institutional service with a clear upgrade roadmap and evaluation indicators, enabling the infrastructure to support growing national and regional research programs. A detailed UVRI use-case illustrating this rule is provided in supplementary section S1.10.

## 5. Applications in bioinformatics

The UVRI cluster was purpose-built to address the growing computational and data management demands of genomics and bioinformatics research in Africa. Since its deployment, it has become a key resource enabling scientific discovery, workforce development, and health systems strengthening. We describe three representative use cases that highlight its direct scientific impact.

### 5.1. Emerging and re-emerging pathogen surveillance

The HPC infrastructure at UVRI has supported a range of viral genomics applications enabling early detection and monitoring of emerging and re-emerging viral pathogens in Uganda which is key in outbreak preparedness and response. It powered the analysis of SARS-CoV-2 genomic data to track viral evolution, including studies that documented the rapid replacement of earlier lineages by the Delta variant and the subsequent emergence of Omicron (Bbosa et al. 2022). The platform has also been instrumental in pathogen surveillance efforts. In 2023, metagenomic analyses conducted on the cluster identified anthrax as the cause of a previously unexplained deaths in Uganda (Bbosa et al. 2025). Similarly, it enabled assembly of Mpox virus genomes from the first confirmed Mpox cases during the 2024 outbreak in Uganda (Bbosa et al. 2025). Beyond outbreak response, the platform has supported researchers working on HIV vaccines studies to conduct structural modelling to identify clade-specific HIV-1 in African children (Kyobe et al. 2024). The platform has been used for development of portable tools for analysis of next-generation sequencing (NGS)-based HIV drug resistance testing data (Ssekagiri et al. 2022, 2024). Typical workloads consist of 16–64 parallel CPU cores per job, consuming 64–256 GB of memory per workflow and generating upto 0.5TB of intermediate data per batch, Automation via Nextflow pipelines and Slurm job arrays ensures scalability and traceability. Data are stored in tiered storage with primary storage for active analysis and automated backup to external disks for long term retention.

### 5.2. Malaria molecular surveillance

The cluster is routinely used to analyze targeted deep sequencing data generated for the molecular surveillance of malaria. These include investigations of insecticide resistance markers based on *Anopheles gambiae s.l.* amplicons (Nagi et al. 2025). An automated pipeline co-developed with colleagues at LSTM using Snakemake, papermill and Jupyterbook manage tasks such as demultiplexing, read alignment, variant calling and rendering of an analyse book used to generate results. These analyses are intended to inform national malaria control strategies and are integral to ongoing genomic surveillance activities in East Africa. Amplicon-based and whole genome analyses of malaria vector studies use Snakemake and Nextflow based workflows integrated with JupyterBook for reporting. Jobs scale across 32—512 cores and leverage Conda recipes and Singularity containers for environment reproducibility.

### 5.3. Data science initiatives

Among several data science initiatives, of unique value is the testing and implementing GA4GH standards as part of a multi-site collaboration involving UCT (South Africa), ACE-Uganda, ACE-Mali, and UVRI (https://github.com/elwazi/elwazi-pilot-node-install). The aim is to set up three interconnected servers that mirror production environments at each site. We’re currently working with three core GA4GH standards: Data Connect, DRS, and WES with plans to integrate passports for access control later. For now, access is managed using firewall rules that restrict traffic to just the participating nodes. As a use case, the ACE2 region was selected from the 1000 Genomes Project CRAM files, indexed them and split the dataset into four batches. Each batch is hosted on a DRS server corresponding to a partner site. A central Data Connect server holds access metadata for all CRAM files across the four DRS servers. Users can query this server using fields like sample ID, population group, super population group and sex and then submit selected CRAMs to a WES endpoint for analysis. The WES workflow processes the input and generates a combined MultiQC report. Our cluster hosts mirror nodes for federated data analysis with each node running containerized services (Data Connect, DRS, WES) deployed using automated scripts. This demonstrates portability to distributed environments while maintaining FAIR and secure access principles.

These real-world applications illustrate our cluster’s foundational role in advancing bioinformatics-driven research, generating actionable insights and supporting the development of computational genomics capacity in Africa.

## 6. Conclusion

Building an HPC cluster in a resource-limited setting requires more than just hardware, it requires a clear strategy, collaboration and a commitment to sustainability. The ten rules presented in this article are derived from our experience at UVRI. These rules encapsulate the essential strategies and practices that have enabled us to overcome challenges and achieve success in a resource-constrained setting while offering a practical roadmap for institutions undertaking similar projects. Each rule emphasizes a critical aspect of the process from understanding the importance of a clear identity to the value of partnerships, training and sustainability. The ten rules presented here distill both technical and organizational lessons from our experience at UVRI into a reproducible and transferable framework. By combining automation, structured service management and explicit risk mitigation strategies, institutions can move beyond ad hoc deployments toward sustainable, standards-aligned HPC operations. From the outset, investing in people, leveraging collaborations and utilizing open-source tools are essential steps in ensuring the success of an HPC cluster. Understanding the environment, strategic planning and defining the scope of operations help to mitigate risks and avoid common pitfalls. Embedding automation and reproducibility through infrastructure-as-code, role based training and documented processes ensures continuity and resilience, even under constrained resources.

What’s clear is that building an HPC cluster is a marathon, not a sprint. By starting small, scaling gradually and continually engaging with stakeholders, resource-constrained institutions can build resilient and effective HPC environments that serve their research goals. Sustainability, both financial and operational, must be considered at every stage. Incorporating transparent documentation, risk registers and open dissemination of configuration templates further strengthens community learning and long term viability. A well-thought-out sustainability plan that includes capacity building, partnerships and institutional buy-in will ensure that the HPC infrastructure remains relevant and impactful for the foreseeable future. We hope that the insights shared in this article will inspire and guide other institutions as they navigate the complex yet rewarding multifaceted journey of building an HPC cluster. With the right approach, even in resource-limited settings, HPC can become a powerful tool for advancing research and innovation.

## Supplementary Material

btag149_Supplementary_Data

## Data Availability

All data underlying this article are available in the manuscript and its online supplementary material.
