# Supplementary material for: A scalable HPC framework for bioinformatics in resource-limited settings: design principles, implementation, and sustainability from the UVRI experience
Source: Bioinformatics. 2026 Mar 25;42(4):btag149. doi: 10.1093/bioinformatics/btag149 (PMC13076007; doi:10.1093/bioinformatics/btag149)
Supplement: btag149_Supplementary_Data [file btag149_supplementary_data.docx]

**Supplementary data**

*(Accompanies the manuscript: “A scalable HPC framework for bioinformatics in resource-limited settings: design principles, implementation and sustainability from the UVRI experience”)*

**S0. Overview**

This supplementary data file provides expanded descriptions of the UVRI use cases referenced in the main manuscript. These examples offer additional technical detail, context and background that illustrate how the general framework and ten rules were applied in practice.

**S1. Use-case for each rule**

**S1.1 Rule 1: Invest in people from within the ranks where possible**

Our team participated in various training opportunities to nurture their expertise and align with global HPC trends. Our team’s first hands‑on training started where every HPC journey begins, inside Linux. Owing to the institute’s sponsorship to Linux workshops and a significant dose of self‑study, we spent weeks tinkering with user management, job schedulers, shared storage and basic monitoring on a sandbox of compute nodes, slowly demystifying the moving parts of a production‑grade system. The real breakthrough, though, came when we plugged into the quarterly virtual workshops for system administrators hosted by the HPC Ecosystems project ^1^ at the Centre for High‑Performance Computing ^2^ (CHPC) in South Africa. These workshops (https://events.chpc.ac.za/event/130/) provided hands-on experience with deploying a virtual cluster using the OpenHPC2.x software stack. Participants gained essential skills, including parallel computing concepts, compiling software and deploying a fully functional OpenHPC management server which could be migrated to a physical system. This program is delivered in a virtual classroom environment which allowed our staff to participate asynchronously, fitting neatly around their own time constraints and workloads. In addition, our team took the CS6290: High-Performance Computer Architecture course ^3^ offered by the Georgia Institute of Technology as part of their Online Master of Science in Computer Science (OMSCS) program. This course covered advanced topics such as branch prediction, out-of-order execution, multi-level caches, and many-core processors. It equipped our team with the knowledge to optimize both software design and hardware architecture for improved performance. Investing in people has allowed us to build a team capable of adapting to new challenges and innovations to ensure long-term operational stability and growth.

**S1.2 Rule 2: Leverage collaborations**

Northern institutions regularly upgrade their equipment, leaving behind used but still powerful machines that could be extremely valuable to institutions in the South. We experienced this firsthand when we received servers from the Francis Crick Institute through the MUII program. These were a major upgrade to our infrastructure, forming the foundation of our cluster without the massive upfront costs of new equipment.

Notably, our bioinformatics infrastructure journey began with startup capacity provided by H3ABioNet. Additionally, they invested in critical upgrades to enhance the HPC's performance and capacity to effectively handle the growing demands of genomic research in our ecosystem. However, collaborations are about more than receiving hardware donations. The long-term value of partnerships lies in the exchange of knowledge and expertise. For example, we leveraged our partnership with the H3ABioNet through the infrastructure working group which provided valuable insights into how to design, manage and sustain HPC infrastructure in environments like ours. Their shared experiences, best practices and technical support was instrumental in addressing cluster configuration, software management and end-user support. We adopted consortium-wide guidance developed by H3ABioNet on optimal bandwidth requirements, which proved critical in equipping our infrastructure to effectively support large-scale genomic data collaborations. We also established a close relationship with the HPC Ecosystems project, an initiative that focuses on facilitating the deployment of HPC across Africa and providing training for HPC system administrators. Through this connection, UVRI gained access to vital technical support and training resources including quarterly virtual workshops on OpenHPC deployment and system management. These collaborations extended beyond immediate technical support. They provided us with access to communities who were facing or had already overcome the challenges we were grappling with. Additionally, these partnerships helped us navigate potential pitfalls by learning from the experiences of others. Partnerships with more advanced institutions turned what could have been a daunting resource-heavy endeavour into a manageable scalable success.

**S1.3 Rule 3: Go open source**

One of the most significant advantages of open-source software is the community behind it. These communities are constantly evolving, with new tools being developed, tested and improved by a global network of users and contributors. After weighing our options, we opted for OpenHPC ^4^, an open-source platform that integrates various management and user tools into a comprehensive modular HPC solution. The decision was largely based on the extensive community support, ongoing development and the modularity of the OpenHPC framework. The OpenHPC community is particularly active in Africa through initiatives like the HPC Ecosystems Project. OpenHPC allowed us to build and manage a system that meets our immediate needs while providing the flexibility to scale as our research demands grew.

Beyond OpenHPC, we also embraced other open-source tools that support the broader HPC ecosystem: a) Containerization - For containerized computing, we adopted Singularity ^5^ to run applications in isolated environments. b) Monitoring the health and performance - we chose Ganglia ^6^ to monitor the cluster in real time. Its lightweight nature and scalability made it ideal for our setup, providing a clear view of system load, network traffic, and resource utilization. c) To monitor usage patterns, benchmark performance and ensure the cluster was being utilized effectively, we deployed XD Metrics on Demand ^7^ (XDMoD). This tool gives us insights into how users are engaging with the cluster and helps us allocate resources better. Together these components form a transparent, auditable stack that aligns with FAIR and open science principles.

The beauty of open-source software lies in the collaborative environment it fosters. Being part of the OpenHPC community provided us with a wealth of knowledge, troubleshooting support and a continuous stream of updates that allowed our system to evolve with the latest advancements in HPC management. The support from such communities was particularly invaluable given our limited internal IT resources. Open source not only kept our operational costs low but also provided access to global expertise and innovations we would not have been able to achieve on our own.

**S1.4 Rule 4: Plan accordingly**

One of the first major challenges we faced was ensuring we had the right environmental infrastructure to support the cluster. HPC systems are power-hungry and generate a significant amount of heat which can lead to overheating and hardware failure if not managed properly. We are located in a region where power fluctuations and outages are common thus needed to make sure we had a reliable power supply including backup solutions before deploying our cluster. This involved setting up a solar-powered battery bank solution with two input feeds; the mains and the solar to ensure the cluster could continue running uninterrupted during power cuts but also with Uninterruptible Power Supply (UPS) functionality to protect the cluster from voltage surges. Cooling was another major consideration. Servers generate significant heat and without proper cooling systems, the risk of overheating could degrade the performance or even damage the equipment. Our planning included assessing airflow in the server room, calculating the cooling load and subsequently implementing a redundant robust cooling system to ensure that if one unit failed, another would take over.

Given the intensive data processing needs of genomic research and other computational tasks, having a fast and reliable network was critical to the success of the HPC cluster. Before deploying the servers, we upgraded our internal network infrastructure to support the high-speed data transfers that would be required. This involved installing a 10-gigabit Ethernet network to ensure low-latency communication between nodes. Our planning also took into account the data storage needs of the cluster. Genomic datasets are massive, often reaching terabytes in size. To manage this, we set up a high-performance hot storage system with sufficient capacity to handle current and scalability capacity to accommodate future research data. To ensure no data is lost in case of hardware failure, we have set-up a redundant data backup system. **Bioinformatics projects often involve moving large datasets, sometimes across institutions or nations. We planned for reliable and secure data transfer mechanisms by exploring efficient tools, particularly Globus ^8^ and Aspera ^9^.** HPC storage is expensive and finite, and without proper retention policies, old data can accumulate and strain resources. We are developing data lifecycle management guidelines; defining what should be kept long-term, what can be archived and what must be deleted after a certain period to ensure the system remains usable and sustainable over time.

Another key component of our plan involved ensuring we had the right people with the right skills to manage and operate the HPC cluster. At UVRI, we had IT staff who were proficient in basic system administration but managing an HPC system requires specialized knowledge in cluster management, workload scheduling and parallel computing. We realized early on that we needed to invest in building the necessary human capacity to run the cluster efficiently. To address this, we took advantage of several training opportunities within the global HPC community. Beyond technical training, we also intend to emphasize cross-training within the broader UVRI research community. Our researchers are to be introduced to basic HPC usage, parallel computing concepts and how to submit jobs to the cluster to ensure they make efficient use of the system. Bringing researchers and IT into a shared understanding of the HPC system shall reduce the likelihood of operational bottlenecks and improve overall productivity.

**S1.5 Rule 5: Document everything**

We recognized early on that documentation would be key to the sustainability and long-term success. Our documentation process started during the initial planning phase and continued through deployment, training and daily operations. We created a wide range of materials, including: 1) User manuals - To provide step-by-step guides for end users on how to interact with the HPC cluster. From basic job submissions to advanced parallel computing workflows, the user manuals cater to both novice and experienced users. 2) System administrator guides - To document everything from cluster setup and management to troubleshooting common issues. This documentation is not only invaluable as we onboard new team members so they can get up to speed quickly and independently but also facilitate a smooth handover in the event of staff changes. 3) Policy documents - In addition to technical guides, we developed clear policy documents aligned with FitSM concepts (incident handling, access control, change requests) that govern the usage of the HPC resources. These policies define acceptable use, access privileges, resource allocation and job scheduling priorities. To ensure equitable access to the HPC resources, the policies help users understand their responsibilities and the best practices for using the HPC cluster efficiently. 4) Training materials - To complement our written documentation, we are currently developing a range of training materials including presentations, video tutorials and hands-on workshop content. Making these resources readily available empowers users to resolve issues independently reducing the burden on the IT team. 5) Troubleshooting logs and FAQs - As our team navigated the challenges of deploying and managing an HPC cluster, we encountered various technical and operational issues. Rather than viewing these challenges as setbacks, we saw them as opportunities to build institutional knowledge. We keep a detailed log of issues including configuration errors, network failures, software bugs and hardware glitches. For each issue, we documented the troubleshooting steps taken and the eventual solutions, allowing us to create a comprehensive FAQ and troubleshooting guide. This resource will not only streamline future problem-solving but also contribute to our goal of knowledge sharing within the broader HPC community.

**S1.6 Rule 6: Define your scope**

It's essential to define who your target users are and which types of projects you will support. For UVRI, this meant identifying research areas that would benefit the most from HPC and ensuring that the infrastructure served those needs without spreading resources too thin. The cluster was designed to serve the following primary user groups and research areas: 1) Genomics and bioinformatics research - the primary users of the UVRI HPC cluster are researchers working on genomics and bioinformatics, particularly those involved in high-throughput sequencing data processing and analysis. This includes genome-wide association studies (GWAS), whole-genome sequencing (WGS), transcriptomics (RNA-seq) and variant calling for pathogen genomics. In addition to pathogen genomics, the HPC cluster also supports research in Malaria molecular surveillance (MMS) particularly in vector genomic investigations such as assessing population structure, identification of cryptic species and investigation of spread of mutations/ or variants conferring insecticide resistance. This work, which contributes to the growing field of vector control by genomic insights, is an under-serviced area that adds significant value to the cluster’s impact in public health research in the context of Malaria.

The scope extends to processing complex bioinformatics workflows that require intensive computation. 2) Infectious disease research - As UVRI is a key player in infectious disease research, the HPC cluster supports epidemiological and pathogen studies with a particular focus. 3) Collaborative regional research initiatives – This infrastructure also supports external collaborative research initiatives. These include eLwazi ^10^, PANGEA ^11^, East African Vector Surveillance project - a joint UVRI, Kenya Medical Research Institute, Wellcome Sanger Institute and Liverpool School of Tropical Medicine research collaboration on embedding genomic surveillance into vector control trials and interventions where UVRI contributes computational resources to large-scale region-wide studies on population genetics, genomics of disease vectors and surveillance data. UVRI leverages the cluster to support partner institutions that may not have local compute resources particularly for collaborative analyses and bioinformatics capacity-building efforts. This includes projects where data sharing and multi-institutional analysis are key components of the research. 4) HPC education and training - The cluster also serves as a training platform for building local capacity in computational biology, bioinformatics, and HPC system administration. Through partnerships and regional collaborations, the cluster can support hands-on training sessions for bioinformatics students and early-career researchers in using HPC for biological data analysis and technical workshops for IT staff in HPC administration. 5) Data-driven public health research - With the rise of data-driven research in public health, the cluster is set up to also support project that involve large-scale data analyses for public health interventions. Computational modeling of disease outbreaks, health informatics and machine learning-driven public health studies are also supported.

Some of the key considerations in defining our scope included 1) Prioritization of critical research. We focused our computational resources on projects with high public health impact and relevance to Uganda and Africa to ensure the cluster is used efficiently for strategic research areas. 2) Controlled access and fair usage. To prevent the cluster from being overburdened, usage policies were implemented with clear criteria for access, prioritization based on project alignment with UVRI's strategic goals and resource allocation guidelines. 3) Modular expansion. Our scope allows for gradual scaling of the infrastructure. As new projects and collaborations emerge, additional resources can be planned for based on demand and funding availability to ensure the cluster remains flexible but targeted in its purpose.

**S1.7 Rule 7: Take your time**

Taking a measured and patient approach during the HPC cluster deployment process proved essential for ensuring long-term success. We discuss here two specific examples where this approach was applied: 1) Consulting with regional and international experts before configuring the hardware for the HPC cluster. We spent considerable time consulting with the HPC Ecosystems, the H3ABioNet and RSSE Africa ^12^. This provided us with valuable insights into HPC design and deployment best practices for resource-constrained environments. These consultations included advice on hardware, optimal configurations and operational challenges. By taking time to gather this knowledge, we avoided common pitfalls such as selecting incompatible hardware components that could have increased costs and complexity. The result was a tailored infrastructure that fit the institute’s research needs without overshooting its operational capacity. 2) Phased rollout. We chose to adopt a phased rollout for the HPC cluster instead of deploying all available hardware and software at once. Initially, only a small number of servers from the machines donated by the Francis Crick Institute were deployed. This allowed the IT team to familiarize with managing the smaller setup, perform extensive testing and troubleshoot issues before scaling up to full capacity. This slow deliberate approach helped refine the system architecture, optimize resource allocation and ensure proper training for the IT staff in maintaining the system. As a result, by the time we scaled up to full capacity, we had built a stable and reliable foundation for ongoing operations.

**S1.8 Rule 8: Start small, scale gradually**

Here’s how this strategy worked in our case. Phase 1: Basic networking and a small number of nodes - In the initial phase, we deployed a modest setup of 5 nodes with basic networking infrastructure. This allowed us to test the waters by running simple computational tasks and ensuring that the foundational elements such as node communication and data transfer were functioning smoothly. The networking configuration was basic with all management services such as job scheduling and user authentication, hosted on a single node. This compact setup reduced complexity and allowed the IT team to get acquainted with managing the cluster before scaling up. Phase 2: Incremental upgrades as needs evolved - The second phase served as a "lighting phase," where issues and bottlenecks in the system could be identified early. For instance, as more researchers began to use the cluster, we observed challenges with networking speeds due to increased traffic between nodes. In response, we focused on upgrading the networking infrastructure by adding a 10gb ethernet switch and optimizing the network layout to improve data throughput and reduce latency between nodes. This phase also involved fine-tuning the storage setup to accommodate larger datasets, a need identified as users started running more data-intensive workflows. Phase 3: Enhancing computational power and parallel processing - As more complex research projects demanded greater computational power, we recognized the need to upgrade the cluster's processing capabilities. We intend to gradually replace older processors with more advanced multi-core CPUs to improve parallel processing performance. The approach allows us to scale up computational capacity incrementally without overwhelming the existing infrastructure. Phase 4: Introducing GPU nodes for specialized workloads **-** Once we reach a point where researchers need to run computationally intensive tasks involving machine learning and other GPU-optimized algorithms, we shall have entered the GPU phase. Rather than immediately invest in an expensive and fully-fledged GPU setup, the cluster will be expanded with a few specialized GPU nodes. This shall allow the research teams to begin exploring GPU-accelerated computing for specific tasks such as large-scale simulations and image analysis without overburdening the cluster with unnecessary hardware.

Each upgrade whether related to networking, CPU performance or GPU capabilities is driven by actual demand to reduce the risk of overinvestment and ensure the system is always aligned with user needs. This phased approach would also give the IT team the space to learn and adapt, making sure that when the time comes for each new phase, they have the experience and expertise to implement it effectively. Ultimately, this strategy results in a robust scalable HPC infrastructure that evolves as research needs and technical challenges grow over time.

**S1.9 Rule 9: Maintain flexibility in infrastructure and operations**

Here are two key examples of how flexibility was implemented: From the outset, our HPC setup was designed with modularity in mind. Rather than committing to a rigid large-scale infrastructure from the start, we selected modular components that allowed for seamless upgrades. For instance, when we initially deployed the cluster, we started with a few nodes using basic networking. As the demands on the system grew—such as increased data processing and more users—we were able to add nodes incrementally, upgrade networking infrastructure, and expand storage capacity without needing to redesign the entire system. This modular approach meant we could scale up as needed without disrupting ongoing research or stretching our budget unnecessarily. When GPU-intensive research begins to emerge, we won’t overhaul the entire infrastructure. Instead, we shall add a few GPU nodes to handle specific computationally heavy tasks like deep learning and image analysis, leaving the rest of the system untouched for standard workloads. This allows us to cater to specialized demands without committing to a large investment in GPUs from the outset. On the operational side, we maintained flexibility by allowing our HPC management processes to evolve over time. Initially, we operated with a single management node that handled job scheduling, user management monitoring and data transfer. As the cluster usage increased and workflow systems like Nextflow ^13^, Snakemake ^14,15^ and WDL^16^ for bioinformatics pipelines were introduced, the operational workflows had to be restructured to accommodate more complex needs. We also remained open to feedback from users and adapted our resource allocation policies and job scheduling systems accordingly. For example, when certain research groups require longer computational runtimes for large genomic datasets, we adjust the job scheduling policies to allow for different queue lengths and resource allocations. This flexibility ensures that the cluster remains useful and relevant to all users despite the diversity in their computational needs.

**S1.10 Rule 10: Have a sustainability plan**

Our long-term sustainability plan translated to a need for a financial model that fits our unique environment. We focused on: 1) A clear pricing model - Drawing from the cost-recovery approaches observed in the benchmarked institutions, we developed a pricing structure for users of the HPC cluster. This was designed to balance affordability with the need to maintain and upgrade the system. One of our key benchmarks was the ACE (Africa Center of Excellence) ^17^ cluster at the Infectious Diseases Institute (IDI) at Makerere University which provided a close-to-home example of an HPC system serving a similar research community in Uganda. The ACE cluster's sustainability plan is built on a robust cost-recovery model.

2) Institutional buy-in - Securing the support of UVRI leadership was key to our sustainability plan, achieved by engaging other institutional groups through a clear development plan and demonstrated capacity which showcased the cluster’s potential to support diverse computational research. By demonstrating the potential impact of the HPC cluster on research output and grant acquisition, we were able to secure both financial backing and administrative support for its ongoing operation. Additionally, we leveraged the HPC resource to attract grants and establish partnerships, staging the cluster as an integral part of UVRI’s broader research ecosystem and future growth.

3) Knowledge management – we also drew significant insights from the CHPC. The CHPC operates on a large scale, serving as a regional HPC hub for various African research institutions but their focus on sustainability through community-building and skills transfer was particularly inspiring. Through the HPC Ecosystems Project, CHPC emphasized not just the deployment of hardware but also the training of HPC professionals in partner institutions to foster long-term sustainability through knowledge transfer. UVRI adopted this approach by investing in the training of system administrators to ensure that local capacity could manage and grow the cluster over time.

4) Long-term planning - Our sustainability plan also accounted for the evolving needs of our user base. As more researchers at UVRI and partner institutions began utilizing the HPC cluster for larger complex projects, we anticipated that demand for compute resources would increase. We structured our pricing and operational plans to be flexible to allow for phased expansion of the cluster based on research needs and funding availability.

**S2. Supplementary References**

1. Johnston, B., Timm, L., Macleod, D. & Poole, J. Ten Years of the HPC Ecosystems Project - Transforming HPC in Africa for the past decade. in *Practice and Experience in Advanced Research Computing 2024: Human Powered Computing* (Association for Computing Machinery, New York, NY, USA, 2024). doi:10.1145/3626203.3670537.

2. Centre for High-Performance Computing (CHPC) https://chpc.ac.za/.

3. High-Performance Computer Architecture course, Georgia Institute of Technology https://omscs.gatech.edu/cs-6290-high-performance-computer-architecture.

4. Baird, R., Schulz, K., Simmel, D., Sundararajan, N. & Hensbergen, E. Getting Started with OpenHPC. Preprint at (2023).

5. Kurtzer, G., Sochat, V. & Bauer, M. Singularity: Scientific containers for mobility of compute. *PLoS One* **12**, (2017).

6. Massie, M., Chun, B. & Culler, D. The Ganglia Distributed Monitoring System: Design, Implementation And Experience. *Parallel Comput* **30**, 817–840 (2004).

7. Palmer, J. T. *et al.* Open XDMoD: A Tool for the Comprehensive Management of High-Performance Computing Resources. *Comput Sci Eng* **17**, 52–62 (2015).

8. Globus. (n.d.). Fast, reliable research data transfer. Retrieved June 2025, from https://www.globus.org/data-transfer.

9. IBM Aspera. (n.d.). High-speed data transfer for the enterprise. Retrieved June 2025, https://www.ibm.com/products/aspera.

10. eLwazi Open Data Science Platform https://elwazi.org/.

11. Pillay, D. *et al.* PANGEA-HIV: Phylogenetics for generalised epidemics in Africa. *The Lancet Infectious Diseases* vol. 15 259–261 Preprint at https://doi.org/10.1016/S1473-3099(15)70036-8 (2015).

12. Research Software & Systems Engineers of Africa https://rsse.africa/.

13. Di Tommaso, P. *et al.* Nextflow enables reproducible computational workflows. *Nat Biotechnol* **35**, 316–319 (2017).

14. Köster, J. & Rahmann, S. Snakemake—a scalable bioinformatics workflow engine. *Bioinformatics* **34**, (2018).

15. Mölder, F. *et al.* Sustainable data analysis with Snakemake. *F1000Res* **10**, 33 (2021).

16. OpenWDL. Workflow Description Language. https://openwdl.org.

17. ACE – African Center of Excellence in Bioinformatics & Data Sciences https://ace.ac.ug.
